# Preoperative radiomic biomarkers reflect functional shoulder impairment in rotator cuff tears: a structure–function analysis

**DOI:** 10.3389/fbioe.2026.1774121

**Published:** 2026-07-06

**Authors:** Martina Sassi, Letizia Mancini, Arianna Carnevale, Matilde Mancuso, Paolo Giaccone, Luca Bacco, Mario Merone, Carlo Casciaro, Emiliano Schena, Ara Nazarian, Alessandro de Sire, Pieter De Hooghe, Rocco Papalia, Gaia Roccaforte, Giovanni Pioggia, Leandro Pecchia, Umile Giuseppe Longo

**Affiliations:** 1 Fondazione Policlinico Universitario Campus Bio-Medico, Rome, Italy; 2 Universita Campus Bio-Medico di Roma, Rome, Italy; 3 Carl J. Shapiro Department of Orthopaedic Surgery and Center for Advanced Orthopaedic Studies, Beth Israel Deaconess Medical Center, Harvard Medical School, Boston, MA, United States; 4 Physical and Rehabilitative Medicine, Department of Medical and Surgical Sciences, University of Catanzaro “Magna Graecia”, Catanzaro, Italy; 5 Research Center on Musculoskeletal Health, University of Catanzaro “Magna Graecia”, Catanzaro, Italy; 6 Department of Orthopaedic Surgery and Sports Medicine, Aspetar Orthopaedic and Sports Medicine Hospital, Doha, Qatar; 7 Institute for Biomedical Research and Innovation (IRIB), National Research Council of Italy (CNR), Messina, Italy; 8 Department of Biomedical, Dental and Morphological and Functional Imaging Sciences, University of Messina, Messina, Italy

**Keywords:** correlation, kinematics, magnetic resonance imaging, optical motion capture systems, radiomics, rotator cuff tears

## Abstract

**Introduction:**

Rotator cuff tears (RCTs) are a major cause of shoulder dysfunction and functional limitation. Although magnetic resonance imaging (MRI) is fundamental for assessing tendon integrity, it provides a static representation that may not fully explain functional impairment. Optoelectronic motion capture systems enable quantitative assessment of three-dimensional (3D) shoulder kinematics. The aim of this study was to investigate the relationship between preoperative MRI-derived radiomic features and shoulder kinematic parameters in patients with supraspinatus tendon tears.

**Methods:**

A total of 73 patients with RCTs underwent preoperative MRI and 3D shoulder kinematic assessment during four dynamic movements. Radiomic features were extracted from MRI images, while kinematic parameters were computed from motion capture data. Correlation analyses were conducted on the entire patient cohort and after stratification by the American Shoulder and Elbow Surgeons (ASES) score to further investigate whether specific clinical characteristics influenced these relationships.

**Results:**

No significant associations were observed in the full cohort. In contrast, multiple moderate radiomic-kinematic correlations emerged in ASES-based subgroups. Both positive and negative associations were identified between shoulder kinematic parameters and radiomic feature categories, predominantly involving texture- and intensity-based features, with additional contributions from shape and general descriptors.

**Discussion:**

By integrating radiomics with dynamic motion analysis, this study shows that MRI-derived tissue-level alterations are associated with real-world functional shoulder performance, reinforcing the value of combining imaging-based biomarkers with functional assessments for a more comprehensive evaluation of shoulder pathology.

## 1 Introduction

Shoulder pain is a widespread condition, estimated to be the third most common musculoskeletal disorder encountered in clinical practice (Lucas et al., 2022; Duan et al., 2021). Rotator cuff tears (RCTs) are a leading cause of shoulder dysfunction, characterized by pain, reduced function, and decreased quality of life (Khadilkar et al., 2014; Bedi et al., 2024; Franceschi et al., 2007; Franceschi et al., 2008). Their prevalence increases significantly with age, affecting up to 70% of individuals over 70 years old (Micallef et al., 2019; Schnorenberg et al., 2022; Longo et al., 2017). Objective and validated functional assessments are important for supporting diagnosis, guiding treatment, and monitoring the progress of patients with RCTs (Longo et al., 2021; Longo et al., 2011). Nevertheless, despite recent advances, the relationship between structural damage and functional impairment remains poorly understood. A well-recognized challenge in shoulder assessment is the discrepancy between structural findings and functional performance. Patients with comparable imaging findings may exhibit markedly different levels of function, with some maintaining good movement despite substantial structural alterations, while others show significant impairment in the presence of less severe lesions. This mismatch suggests that structural evaluation alone may not fully capture clinically relevant aspects of shoulder function. Patients with comparable tear characteristics or similar levels of pain may exhibit heterogeneous functional profiles, underscoring the complexity of linking morphological findings to functional outcome (Dunn et al., 2014).

Clinical assessment of shoulder function typically relies on standardized questionnaires and functional scales (Romeo et al., 2004). These encompass general health-related quality of life measures, health utility evaluations, and joint- or condition-specific measures. For instance, the Simple Shoulder Test, the Disability of the Arm, Shoulder and Hand questionnaire, and the ASES test (American Shoulder and Elbow Surgeons) (Longo et al., 2011; Prinja et al., 2021). While these clinical scales are easy to administer and do not require complex equipment, they are inherently subject to operator-dependent variability and subjective interpretation (Maffulli et al., 2011). Clinical evaluations rely on the examiner’s expertise and interpretation, often resulting in a general and qualitative evaluation rather than a precise and objective assessment of shoulder function.

Currently, diagnostic imaging plays a crucial role in identifying and assessing the severity of rotator cuff injuries (Dharshika and Babu, 2024). Magnetic resonance imaging (MRI) is particularly valuable for assessing the severity and specific characteristics of rotator cuff pathology (Dharshika and Babu, 2024; He et al., 2025). In this context, radiomics is an emerging field that extracts high-dimensional quantitative features from medical images, providing accurate, objective information on tear characteristics, such as thickness, size, location, and degree of retraction (He et al., 2025). In recent years, radiomics has been widely applied to improve the prediction of clinical outcomes through non-invasive approaches.

However, conventional MRI still depicts the shoulder in a static and unloaded position, a limitation that may contribute to the mismatch between structural findings and the persistence of pain or functional impairment in many patients (Tempelaere et al., 2016).

This limitation highlights the importance of integrating imaging with dynamic and quantitative motion assessments, such as three-dimensional (3D) kinematic analysis, to understand how structural alterations lead to movement dysfunction.

Optoelectronic motion capture systems are considered the gold standard for analysing human joint kinematics due to their high measurement accuracy. These systems enable the acquisition of complete three-dimensional kinematic data of the shoulder, offering detailed and reproducible insights into joint motion dynamics (Coley et al., 2007). These objective measures of patient motor performance are critical for monitoring patient progress, assessing treatment efficacy, and designing personalized rehabilitation protocols.

Kinematic features provide complementary information and a broader understanding of patient status by objectively quantifying joint function. Analysed together, MRI-based radiomic data and kinematic data can help elucidate the underlying relationship between tissue integrity and movement quality. Therefore, the objective of this exploratory study was to investigate the relationships between preoperative MRI radiomic features and preoperative shoulder kinematic parameters in patients diagnosed with supraspinatus tendon lesions. Identifying significant associations between these two domains may contribute to a more comprehensive understanding of the patient’s overall clinical status by integrating structural imaging biomarkers with functional motion analysis.

## 2 Materials and methods

### 2.1 Demographics

A prospective study was conducted among patients diagnosed with rotator cuff between August 2023 and October 2024 at the Fondazione Policlinico Universitario Campus Bio-Medico in Rome. Patients were then selected according to predefined inclusion and exclusion criteria. Inclusion criteria comprised a full-thickness supraspinatus tendon tear as documented by MRI, absence of prior surgical intervention on the affected shoulder, no history of shoulder instability, and an age exceeding 40 years. Conversely, exclusion criteria included inability or refusal to provide informed consent, prior surgical procedures, and the presence of fractures or additional pathological conditions in the affected shoulder. A total of 143 patients were screened, and 73 (49% females and 51% males) were eligible and included in the final analysis. Table 1 summarizes the demographic characteristics of the patient population. To ensure patient confidentiality and comply with data protection regulations, all personal identifiers were removed from the clinical data and image metadata. Specifically, patient names were deleted, and each data set was assigned a unique consecutive number for identification purposes. The study was carried out in accordance with the Declaration of Helsinki and received approval from the local Ethics Committee (protocol code: CARE-RC).

**TABLE 1:** Demographic characteristics of participants.

Clinical features	Overall	Female	Male
Number of participants	73	36	37
Pathological side	55 R; 18 L	28 R; 8 L	27 R; 10 L
Age (years): mean (SD; range)	59.63 (7.26; 30)	59.86 (7.27; 28)	59.40 (7.35; 28)
Height (cm): mean (SD; range)	167.66 (9.03; 40)	162.19 (6.84; 28)	172.97 (7.85; 32)
Weight (kg): mean (SD; range)	75.03 (16.20; 71)	69.54 (14.93; 66)	80.38 (15.76; 69)
BMI (kg/m^2^): mean (SD; range)	26.53 (4.63; 24.24)	26.37 (5.28; 24.24)	26.69 (3.97; 19.37)

Abbreviations: R = Right. L = Left. SD, Standard deviation. Range = maximum value minus the minimum value. BMI, body mass index.

### 2.2 Clinical outcome and MRI

All participants included in the study were administered the ASES score as a clinical outcome scale (Agel et al., 2023). ASES is an 11-item instrument designed to evaluate the quality of life in patients with shoulder disorders. It comprises two subscales: a functional subscore based on 10 items (each rated from 0 to 3, where 0 indicates maximal dysfunction and 3 indicates normal function) and a pain subscore based on a single item (rated from 0 to 10, where 0 represents no pain and 10 represents the most severe pain). Each subscore accounts for half of the final ASES score, which ranges from 0 (indicating the poorest shoulder function) to 100 (indicating optimal shoulder function). Subsequently, participants were stratified into three levels according to their total ASES score. Individuals with a score below 30 were assigned to level 0, those with scores between 30 and 50 were assigned to level 1, and those with scores above 50 were assigned to level 2 (Mukadam et al., 2024) (Table 2). In addition to clinical assessment, all participants underwent preoperative shoulder MRI on a 1.5 T scanner.

**TABLE 2:** Clinical characteristics.

ASES score	Overall	Female	Male
Level 0	27 (37%)	15 (41.7%)	12 (32.4%)
Level 1	27 (37%)	13 (36.1%)	14 (37.8%)
Level 2	19 (26%)	8 (22.2%)	11 (29.7%)

### 2.3 Kinematic data acquisition

An objective preoperative evaluation of the shoulder was performed for all patients. The evaluation involved executing four dynamic movements of clinical relevance for the evaluation of shoulder functionalities level: Task 1) arm elevation up to about 90° in the sagittal plane (flexion/extension); Task 2) arm elevation up to 120° in the scapular plane (scaption); Task 3) arm elevation up to about 90° in the frontal plane (abduction-adduction movement); Task 4) internal/external rotation with the shoulder at 0° of adduction and the elbow flexed at 90°. Each patient performed five consecutive repetitions of each movement. Those movements were recorded using Qualisys™ Optical Motion Capture System (Qualisys AB, Gothenburg, Sweden), consisting of 10 Miqus M3 cameras (sampling frequency, 100 Hz) and 2 Miqus Videos (sampling frequency, 25 Hz) properly positioned around the perimeter of the room (Mancini et al., 2025). A total of twenty-two photo-reflective markers (diameter, 8 mm) were placed bilaterally to anatomical landmarks of the thorax, upper arms, and forearms, in accordance with the recommendations of the International Society of Biomechanics (Wu et al., 2005). Additionally, clusters of markers were placed on the thorax and bilaterally on the upper arms, forearms, and scapulae (Warner et al., 2015).

### 2.4 MRI segmentation and radiomic feature extraction

**FIGURE 1:**
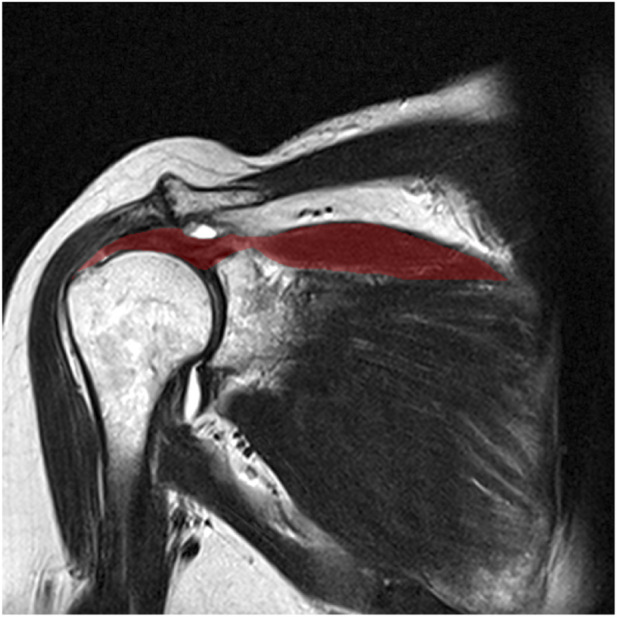
Manual segmentation of the supraspinatus muscle (shown in red) on a T2-coronal MRI slice using ITK-SNAP.

A manual segmentation of the supraspinatus muscle was performed by two expert operators (CC and UGL) on T2-coronal MRI images using ITK-SNAP, delineating the volume of interest (VOI) for each patient (Figure 1) (Yushkevich et al., 2006). To reduce intensity-related variability and improve comparability across scans, image intensity normalization was applied. Specifically, z-score normalization was performed within each VOI by subtracting the mean voxel intensity and dividing by the standard deviation prior to radiomic feature extraction. A total of 112 features were extracted for each 3D VOI using the PyRadiomics open-source software (see Table 3) (PyRadiomics, 2026; Van Griethuysen et al., 2017). Radiomic features were extracted using the default PyRadiomics settings. Gray-level discretization was performed using the default fixed bin width of 25, and no voxel resampling was applied prior to feature extraction. Before feature extraction, image and mask metadata, including spacing, origin, and direction, were verified and harmonized when necessary to ensure spatial correspondence between the image and the VOI mask. Specifically, the following features were extracted: 18 first-order intensity-based statistics, such as mean, 25th, 50th, 75th percentiles, skewness, kurtosis, intensity kurtosis, and intensity variance; 24 derived from the gray level co-occurrence matrices (GLCM); 14 derived from the gray level dependence matrix (GLDM); 16 derived from the gray level run length matrices (GLRLM); 16 derived from the gray level size zone matrix (GLSZM); 5 derived from the neighbouring gray tone difference matrix (NGTDM); 14 shape-based; 5 related to information of the image and mask. All these features were subsequently categorized into four main categories, as summarized in Table 3 (Mayerhoefer et al., 2020). Intensity-based features described the distribution of voxel values within the delineated VOI, providing a statistical characterization of the gray-level histogram of the supraspinatus muscle. Texture-based features, derived from several texture gray-level matrices, quantified spatial patterns and relationships among voxel intensities, offering higher-order statistical information on tissue organization; parameters such as entropy, energy, contrast, and homogeneity captured complex properties including heterogeneity, regularity, and microstructural organization (Scapicchio et al., 2021). Shape-based features reflected the two- and three-dimensional geometry of the VOI, with descriptors such as volume, sphericity, elongation, and flatness providing insights into the selected image area’s physical dimensions and shape characteristics. Finally, general information features included contextual descriptors of the image and mask, such as minimum, maximum, and mean voxel values. To ensure reproducibility and standardization of the extracted features, all features were normalized between 0 and 1 using min-max scaling as a preprocessing step.

**TABLE 3:** Extracted radiomics features categorized into four groups.

Group	Type	Count	Percentage (%)
Intensity	First-order intensity-based statistics	18	16.07
Texture	GLCM	24	21.43
	GLDM	14	12.5
	GLRLM	16	14.29
	GLSZM	16	14.29
	NGTDM	5	4.46
Shape	Shape-based	14	12.5
General	Information of the image and mask	5	4.46

### 2.5 Kinematic feature processing and extraction

The raw motion capture data were preprocessed using the Qualisys Track Manager software for marker trajectories tracking, gap filling and filtering (4th-order Butterworth low-pass filter with a 6 Hz cut-off) (Palermo et al., 2018). Then, the preprocessed data were imported into Visual3D software (C-motion, Germantown, MD, United States) for model definition and shoulder kinematic analysis.

A custom pipeline was developed to segment the upward phases of the humerothoracic joint angles. The onset of each phase was detected from the velocity profiles using a threshold set at 5% of the maximum absolute velocity, while the maximum joint angle marked the end of the phase (Palermo et al., 2018). Of five repetitions, the central three were analyzed to reduce variability.

From these data, two quantitative features were extracted as objective indicators of shoulder mobility, namely, the range of motion (ROM), defined as the difference between maximum and minimum joint angles, and peak angles (maximum angles). For each task, the mean ROM and peak values were calculated across the three repetitions.

### 2.6 Reproducibility and correlation analysis

Inter-operator agreement for the spatial overlap of the ROIs was evaluated using the Dice Similarity Coefficient (DSC) (Dice, 1945), a widely used metric for assessing segmentation reproducibility (Zou et al., 2004). Its value can range from 0 (meaning no spatial overlap) to 1 (indicating perfect overlap). Agreement was interpreted as follows: <0, no agreement; 0–0.2, slight agreement; 0.2–0.4, fair agreement; 0.4–0.6, moderate agreement; 0.6–0.8, substantial agreement; and 0.8–1.0, almost perfect agreement (Pajula et al., 2012; Landis and Koch, 1977).

The statistical relationship between individual MRI-derived radiomics features and shoulder kinematic parameters was investigated, aiming to identify potential associations between structural imaging data and functional performance. Firstly, the distribution of both radiomics and kinematic features was examined using the Shapiro-Wilk test to evaluate normality, with a significance p-value threshold set at 0.05. Depending on the normality results, either parametric (Pearson) or non-parametric (Spearman) correlation analysis were performed to investigate the statistical relationship between MRI-derived radiomics features and kinematic features.

A correlation coefficient of 0.41 was considered as the minimum threshold for a meaningful correlation. The strength of correlation was classified as moderate (0.41–0.60), good (0.61–0.80), or excellent (0.81–1.00) (Mancini et al., 2025; Leong et al., 2022; B et al., 2013). Statistical significance was defined as a p-value <0.05.

This analysis was conducted on the entire patient cohort. To further investigate whether specific clinical or radiological characteristics influenced these relationships, additional subgroup analyses were performed. The dataset was stratified according to the ASES score levels, and correlation analyses were then repeated within each subgroup. Only subgroups containing more than five subjects were considered to ensure adequate statistical power.

This analysis was conducted on the entire patient cohort. To further investigate whether radiomic–kinematic relationships varied across clinically distinct functional profiles, additional subgroup analyses were performed. We hypothesized that the relationship between structural imaging features and functional kinematics might differ according to the level of functional impairment, and that such heterogeneity could obscure meaningful relationships when considering the cohort as a whole. Therefore, the dataset was stratified according to the ASES score levels, and correlation analyses were then repeated within each subgroup. Only subgroups containing more than five subjects were considered to ensure adequate statistical power.

## 3 Results

Inter-operator agreement was high, with a median DSC score of 0.94 (IQR: 0.90–0.97), indicating almost perfect agreement and supporting the reliability of the extracted radiomic features.

**FIGURE 2:**
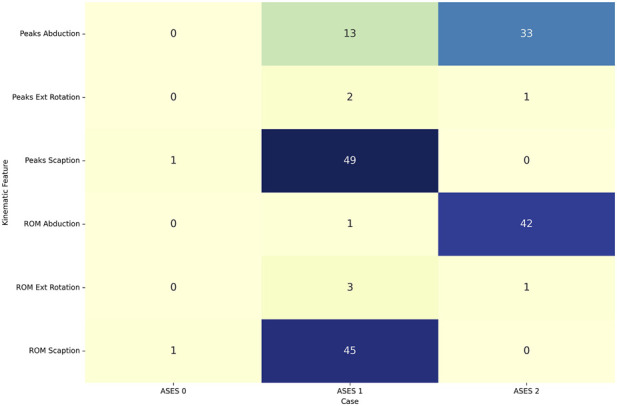
Heatmap illustrating the number of radiomic features significantly correlated to each kinematic variable in all the conducted group analyses.

Since the assumption of data normality for the feature distributions was rejected by the Shapiro-Wilk test, Spearman correlation coefficient was employed to evaluate the relationships between the two variables. Figure 2 presents a heatmap illustrating the number of statistically significant correlations identified between individual kinematic features and the entire set of radiomic features.

The full-cohort analysis did not reveal any statistically significant correlations. This lack of significant associations suggests the presence of substantial heterogeneity within the overall population, which may mask subgroup-specific structure–function relationships. Accordingly, several significant associations emerged when the analysis was stratified by ASES score. Most of the significant associations were found within the ASES 1 subgroup, which accounted for the highest number of correlations across multiple kinematic features, suggesting strong and coherent relationships within this group. The ASES 2 subgroup also exhibited numerous associations, mainly between radiomic features and kinematic measures extracted from abduction movements. An exception was represented by the ASES 0 subgroup, which, despite including the same number of patients as ASES 1, showed relatively few significant correlations. Overall, these findings suggested that subgroup-specific physiological or pathological characteristics, rather than sample size alone, may play a critical role in finding meaningful radiomic–kinematic associations. Moreover, compared to the full-cohort analysis, the stratified approach revealed a markedly higher number of significant correlations, emphasizing the value of subgroup-based analyses in uncovering hidden patterns. All correlation heatmaps for each classification subgroup, along with the corresponding correlation values, are provided as Supplementary Material (Supplementary Figures S1–S3).

**FIGURE 3:**
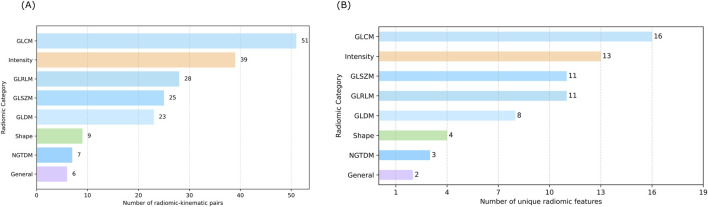
Horizontal bar plots showing the distribution of radiomic feature categories. **(A)** Among the 211 unique radiomic–kinematic pairs. **(B)** Among the 77 unique radiomic features involved.

Across the various subgroup analyses, a total of 192 statistically significant radiomic–kinematic feature pairs were identified. A recurrence analysis was conducted to investigate whether any radiomic–kinematic feature pairs appeared across different clinical subgroups. This analysis revealed 188 unique feature pairs, indicating that only 4 pairs recurred across more than one subgroup, suggesting limited overlap in significant correlations across patient populations. Figure 3A illustrates the distribution of unique feature pairs by radiomic feature category, highlighting the predominance of pairs involving texture-based features. This analysis revealed both feature pairs commonly present across multiple clinical subgroups, potentially indicative of shared underlying mechanisms, and pairs specific to individual subgroups, suggesting possible inter-patient variability in the relationship between tissue structure and biomechanical function.

Since a single radiomic feature can be correlated with multiple kinematic features, a complementary analysis was conducted to identify the unique set of radiomic features contributing to these correlations. Specifically, the proportion of radiomic features within each category that were involved in at least one statistically significant radiomic–kinematic pair was calculated. This analysis identified 68 unique radiomic features, distributed across the radiomic categories, as shown in Figure 3B. A complete list of these 68 radiomic features, along with the frequency with which each appeared in the significant correlations, can be found in the Supplementary Material (Supplementary Figure S4).

**FIGURE 4:**
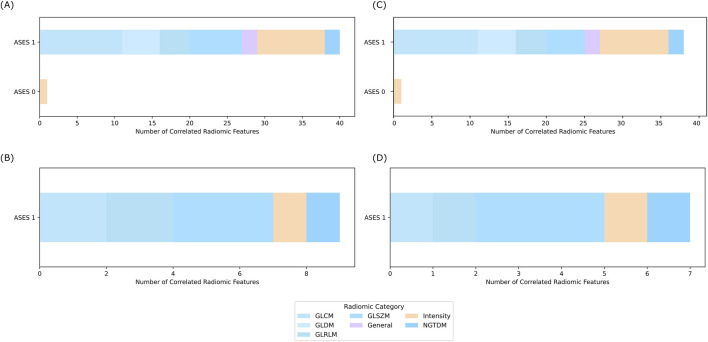
Number of significant correlations between kinematic features extracted from the scaption movement and radiomic categories across clinical cases: **(A)** Positive correlations for the Peak feature; **(B)** Negative correlations for the Peak feature; **(C)** Positive correlations for the ROM feature; **(D)** Negative correlations for the ROM feature.

**FIGURE 5:**
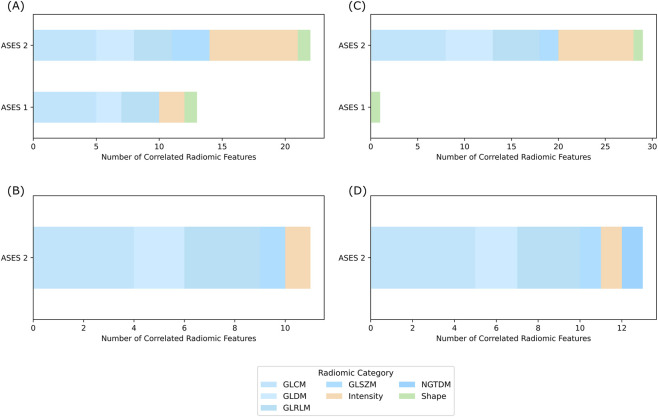
Number of significant correlations between kinematic features extracted from the abduction movement and radiomic categories across clinical cases: **(A)** Positive correlations for the Peak feature; **(B)** Negative correlations for the Peak feature; **(C)** Positive correlations for the ROM feature; **(D)** Negative correlations for the ROM feature.

**FIGURE 6:**
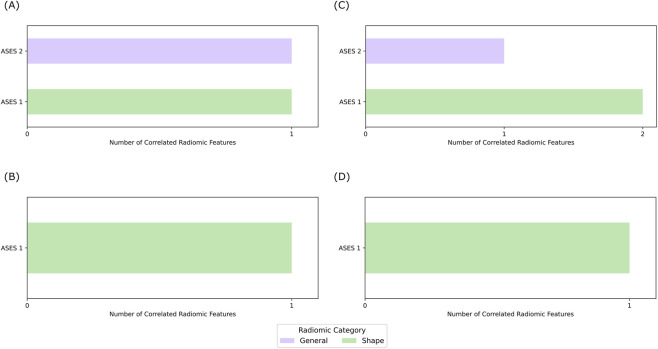
Number of significant correlations between kinematic features extracted from the external rotation movement and radiomic categories across clinical cases: **(A)** Positive correlations for the Peak feature; **(B)** Negative correlations for the Peak feature; **(C)** Positive correlations for the ROM feature; **(D)** Negative correlations for the ROM feature.

To further characterize the radiomic–kinematic associations, the number of unique radiomic features significantly correlated with each kinematic variable was calculated, and results were stratified by radiomic category. This approach enabled the identification of the radiomic categories most frequently associated with specific kinematic parameters, offering insights into the structural domains most relevant to biomechanical function. Figures 4–6 show the number of significant correlations between kinematic features derived from the three movements (scaption, abduction, and external rotation, respectively) and radiomic feature categories. Within each figure, separate panels distinguish correlation direction, with positive correlations displayed in the upper row and negative correlations in the lower row, enabling direct comparison of the sign-specific distribution across radiomic categories. To capture potential heterogeneity among clinical cases, this analysis was stratified by clinical subgroups. This enabled the comparison of radiomic–kinematic association patterns across subgroups, thereby providing a more nuanced understanding of how tissue characteristics relate to joint mechanics under varying clinical conditions.

**FIGURE 7:**
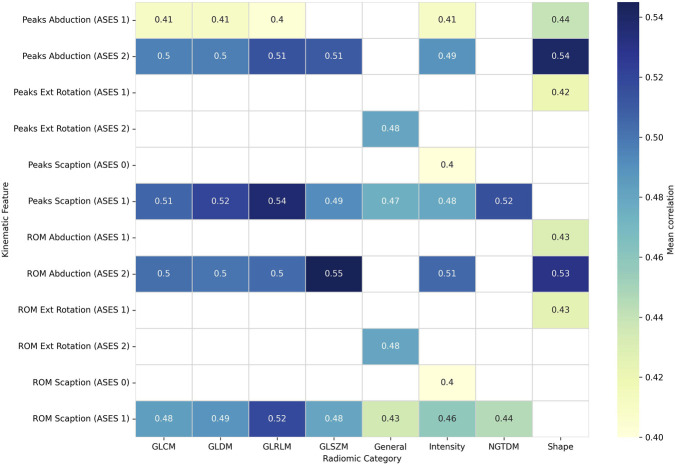
Heatmap of the positive mean correlations between kinematic features and radiomic categories across the different clinical subgroups. Darker shades indicate stronger associations, while grey boxes denote missing values.

**FIGURE 8:**
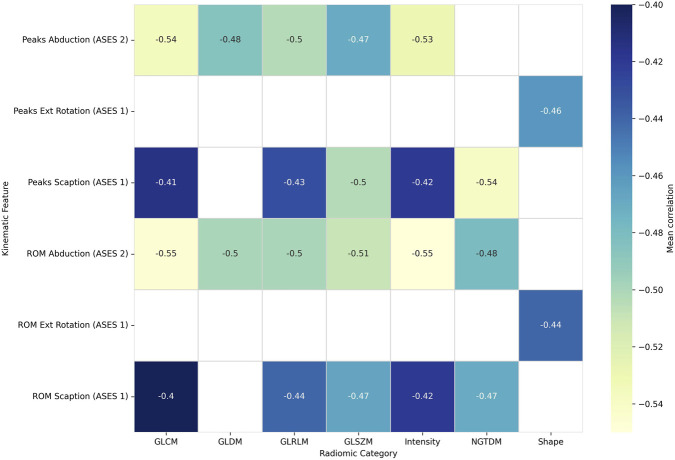
Heatmap of the negative mean correlations between kinematic features and radiomic categories across the different clinical subgroups. Darker shades indicate stronger associations, while grey boxes denote missing values.

Figures 7, 8 show, respectively, the positive and negative correlations between kinematic parameters and radiomic features. For each clinical case, the mean correlation values were computed between every kinematic feature and the set of radiomic features belonging to each category. Each cell in the matrix reports the corresponding mean correlation value, with a colour gradient reflecting its intensity: higher values correspond to darker shades. This visualization provides an immediate overview of which variable combinations exhibit stronger associations and whether these associations tend to cluster within specific clinical subgroups or radiomic categories.

## 4 Discussion

MRI is the gold standard for evaluating the structural integrity of the rotator cuff. Nevertheless, MRI provides a static representation of the shoulder that often does not fully reflect the dynamic limitations experienced by patients. Therefore, complementary approaches, such as three-dimensional kinematic assessment, are increasingly used in clinical practice to provide a dynamic evaluation of patients’ health status. This exploratory study investigated potential correlations between objective kinematic parameters (i.e., ROM and peak values) and radiomic features, aiming to better understand whether kinematic patterns observed during clinically relevant arm movements reflect underlying tissue structure in patients with chronic shoulder pain.

The reliability of the manual segmentation process and the resulting extracted radiomic features is supported by the high inter-operator agreement observed, reinforcing the robustness of the subsequent radiomic–kinematic analyses. While analyses of the entire cohort did not reveal consistent associations, subgroup analyses identified numerous significant correlations. The findings of this study should be interpreted in light of the well-known discrepancy between structural alterations and functional performance. The observed relationships provide preliminary insight into how tissue-level characteristics may relate to movement characteristics, potentially contributing to the understanding of why patients with similar structural findings can present markedly different functional outcomes.

Given the exploratory design of the study, and the large number of correlation analyses performed, which increases the likelihood of false-positive findings, the results should not be interpreted as confirmatory but rather as preliminary observations that may generate hypotheses for future research.

Subgroup analyses highlight the importance of clinical stratification in clarifying structure–function relationships that might otherwise remain hidden within heterogeneous populations. Notably, significant radiomic–kinematic associations emerged only after stratification, whereas no meaningful correlations were detected in the overall cohort. This finding suggests that the increased clinical homogeneity within subgroups may have reduced the masking effect of inter-patient variability, allowing structure–function relationships to become more apparent. However, stratification according to ASES score inevitably reduced the number of subjects within each subgroup, which may have increased the influence of individual observations and reduced the stability of the estimated correlations. Stratified analyses revealed several moderate correlations that varied according to the kinematic variables examined, the radiomic feature categories, and the clinical subgroups considered. Subgroups defined by ASES levels 1 and 2 exhibited the highest number of significant radiomic–kinematic associations. This suggests that in patients with relatively preserved functional capacity, structural imaging biomarkers more closely reflect shoulder biomechanics, likely due to reduced compensatory strategies and less severe pathological changes. Conversely, in the ASES 0 subgroup, characterized by poorer self-reported function, only a few moderate correlations were identified, possibly reflecting higher variability in joint mechanics.

### 4.1 Intensity features

First-order statistics quantify the distribution of voxel intensities and provide the most basic description of tissue composition. In our analysis, features reflecting variability and higher gray-level values, such as *Variance*, *Mean Absolute Deviation*, *Range*, and *Entropy*, showed positive correlations with abduction and scaption.

This pattern was observed across various clinical subgroups. In ASES 1, these features correlated with peak abduction (mean ρ = 0.41), while scaption peak and ROM showed moderate significant correlations (ρ = 0.48 and 0.46). In ASES 2, associations were limited to abduction, with moderate correlations for both peak (ρ = 0.49) and ROM (ρ = 0.51).

Conversely, *Uniformity* was the only first-order feature consistently showing significant negative correlations, and was associated with both peak and ROM scaption in the ASES 1 subgroup (ρ = −0.42) and with peak and ROM abduction in the ASES 2 subgroup (ρ = −0.53 and −0.55). Overall, these findings indicate that higher voxel-intensity heterogeneity is associated with better shoulder function, whereas more homogeneous signal patterns reflect structural compromise and reduced mobility.

### 4.2 Texture features

Texture features capture spatial relationships, repetitiveness, and heterogeneity within the gray-level patterns of the lesion (Scapicchio et al., 2021). Unlike first order features, which quantify only the global distribution of intensity values, texture features describe organizational properties of the tissue microstructure and may therefore provide complementary information about lesion complexity and structural integrity. Across all texture families, significant associations were concentrated in abduction and scaption, mainly in patients with ASES 1 or ASES 2, indicating that these descriptors are most informative when functional capacity is at least partially preserved.

Although each texture family captures a different aspect of spatial organization, a consistent pattern was observed across this category. Features expressing heterogeneity, irregularity or fragmented patterns showed positive correlations with mobility measures. This pattern was particularly evident for GLCM descriptors such as *Cluster Shade* and *Difference Entropy*, in line with previous studies showing the sensitivity of these metrics to microstructural changes (Varriano et al., 2024). Triveni et al. reported that these features detect subtle alterations in patients with shoulder pain, and Scott et al. demonstrated their ability to differentiate healthy from tendinopathic tendons (Triveni et al., 2021; Scott et al., 2024). In our cohort, these heterogeneous texture patterns were most evident in ASES 1 for scaption (peak ρ ≈ 0.49–0.54; ROM ρ ≈ 0.44–0.52) and in ASES 2 for abduction (peak ρ ≈ 0.50–0.51; ROM ρ ≈ 0.50–0.55).

Conversely, features associated with more uniform and low intensity patterns (such as *Inverse Difference Moment*, *Long Run Low Gray Level Emphasis*, *Large Area Low Gray Level Emphasis*, *Busyness*, *Dependence Variance*) showed negative correlations with kinematic parameters. These associations appeared in both ASES 1 (mainly in scaption: ρ ≈ −0.4 to −0.54) and ASES 2 (mainly in abduction: ρ ≈ −0.47 to −0.55), suggesting that microstructural simplification is associated with reduced shoulder function.

These consistent findings strengthen the biomechanical relevance of texture-based radiomic descriptors in capturing structural changes related to joint mobility.

### 4.3 Shape features

Shape features describe the two- and three-dimensional geometry of the lesion and are independent of gray-level intensities. The analysis revealed that elongated lesions extending along their principal axis were associated with better kinematic performance. *Surface Volume Ratio* and *Major Axis Length* showed positive correlations with abduction and external rotation, with moderate associations in ASES 1 (ρ ≈ 0.44 for abduction and ρ ≈ 0.42 for external rotation) and stronger correlations in ASES 2, where *Major Axis Length* reached ρ = 0.54 for peak and ρ = 0.53 for ROM abduction.

Conversely, the Flatness feature, which reflects a more compressed lesion geometry, showed negative correlations. This pattern suggests that elongation along the major axis may support functional preservation, whereas compression along the minor axis may be detrimental. For example, in ASES 1, external rotation peak and ROM were positively associated with *Major Axis Length* but showed a negative mean correlation of −0.45 with Flatness.

### 4.4 General features

General image features represent basic intensity descriptors extracted from the original image, such as the maximum and mean voxel intensity within the lesion, providing simple measures of global signal characteristics. A consistent trend of positive correlations was observed between these features and kinematic parameters derived from scaption and external rotation. Moderate correlations were observed for scaption (ρ = 0.47 for peak and ρ = 0.43 for ROM) within the ASES 1 subgroup, while in the ASES 2 subgroup, both peak and ROM features of external rotation demonstrated moderate correlations of 0.48. Overall, these findings suggest that higher signal intensity values are associated with better preserved joint function, indicating that lesions with brighter or higher-intensity voxels may reflect less structurally compromised tissue, thereby supporting greater mobility.

### 4.5 Strengths, limitations and future perspectives

Overall, our findings support the hypothesis that integrating radiomic features with shoulder kinematics provides complementary information on patient status. To our knowledge, this is the first study to correlate MRI-derived radiomic descriptors with preoperative 3D kinematics in patients with RCT.

The results indicated that greater variability in voxel intensities and more heterogeneous microstructural patterns were associated with better functional performance, whereas uniform or low-complexity signals were associated with reduced mobility. These associations were not limited to texture features, which were predominant, but extended to intensity, shape, and general descriptors, confirming the multidimensional nature of shoulder pathology.

This study presents several strengths. It is, to our knowledge, the first to integrate MRI-derived radiomic descriptors with preoperative 3D shoulder kinematics in patients with full thickness supraspinatus tears, helping to bridge the gap between static structural imaging and functional movement analysis. Notably, while previous radiomics research on the shoulder has largely focused on texture based features, particularly GLCM, our findings highlight the functional relevance of additional radiomic families, including shape, intensity and general descriptors, which have not been previously explored in this context. The use of clinically relevant kinematic tasks and the stratification by ASES score further strengthened the biomechanical interpretation of the results.

Nonetheless, some limitations must be acknowledged. The relatively small sample size, further amplified by stratification into ASES-based subgroups, results in a reduced number of subjects per group, which may decrease statistical power, affect the stability of subgroup-specific associations, and limit the robustness and generalizability of the observed correlations. Furthermore, the present analysis focused exclusively on the associations between radiomic and kinematic features and did not account for potentially relevant clinical and demographic factors, such as age, sex, BMI, tear size, or fatty infiltration, which may influence shoulder function. The cross sectional design also precludes causal inference and does not allow the prediction of postoperative outcomes. These limitations outline clear directions for future research. Larger multicentre datasets, including additional clinical and demographic information, will be crucial for validating the identified associations and improving their statistical robustness, and enabling the application of multivariable models to account for potential confounding factors. Longitudinal studies will be essential to evaluate the predictive value of radiomic descriptors in terms of recovery, retear risk, and rehabilitation trajectories. Finally, the integration of additional biomechanical parameters, such as muscle activation or strength measures, together with the application of multivariable statistical models and machine learning approaches, may further enhance the clinical utility and interpretability of combined radiomic and kinematic assessments.

## 5 Conclusion

This study investigated the relationship between preoperative MRI radiomic features and quantitative, 3D shoulder kinematics in patients with supraspinatus tendon lesions. While MRI is fundamental for defining the structural extent of RCTs, it represents the shoulder in a static and unloaded state, which may partly explain the frequent mismatch between imaging findings and patient reported limitations. By integrating radiomics with dynamic motion analysis, this work highlights how tissue-level alterations captured on MRI relate to real-world functional performance.

Moderate correlations between specific radiomic signatures and kinematic parameters, particularly when stratified by ASES score, suggest that microstructural heterogeneity and geometric preservation are reflected in better movement outcomes. These results reinforce the potential of combining imaging-based biomarkers with functional assessments to provide a more comprehensive evaluation of shoulder pathology.

## Data Availability

The raw data supporting the conclusions of this article will be made available by the authors, without undue reservation.
