# Supplementary figures and images for: Preoperative radiomic biomarkers reflect functional shoulder impairment in rotator cuff tears: a structure–function analysis
Source: Front Bioeng Biotechnol. 2026 Jul 6;14:1774121. doi: 10.3389/fbioe.2026.1774121 (PMC13381298; doi:10.3389/fbioe.2026.1774121)

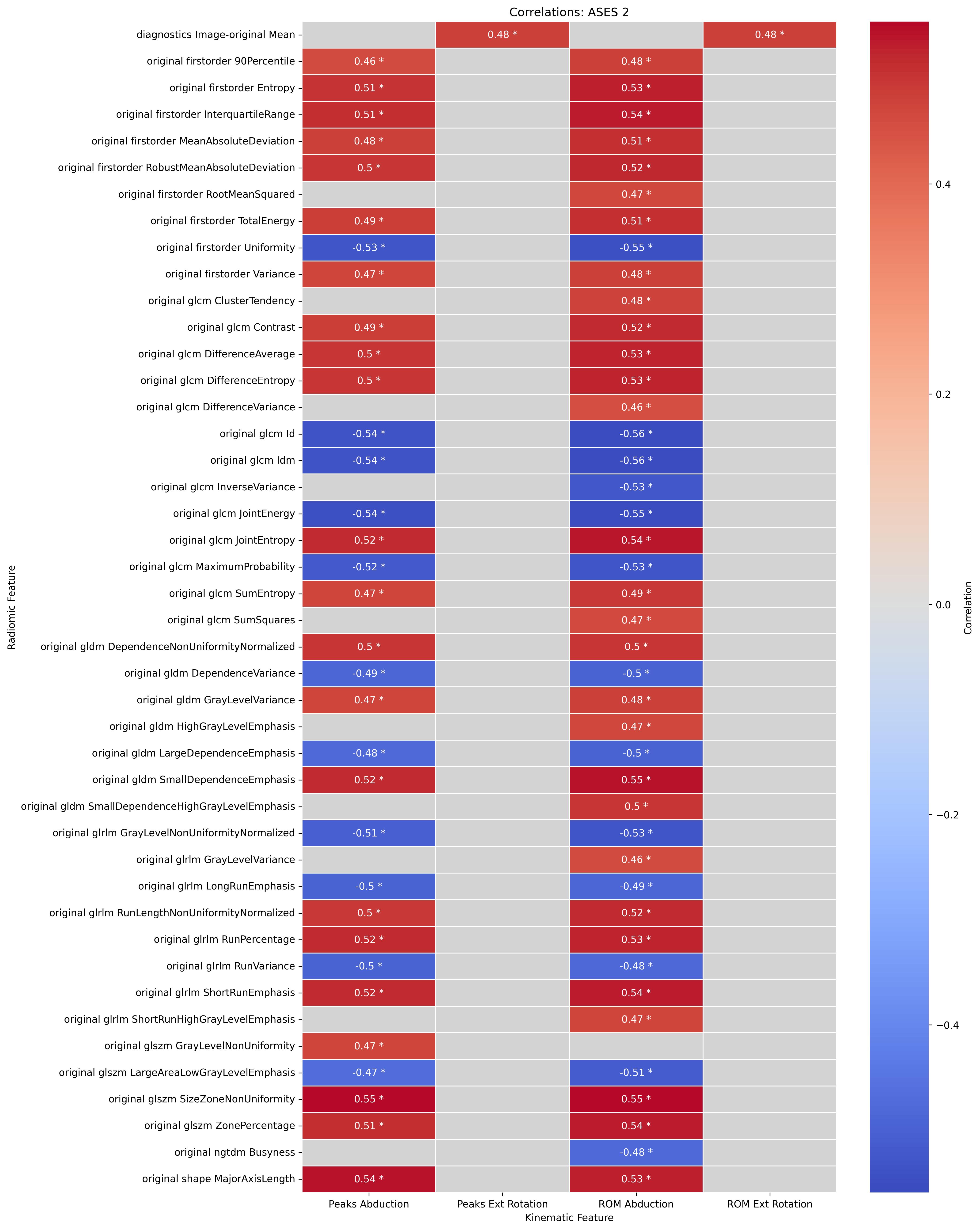

Supplement: Supplementary file 1 [file Image3.JPEG]

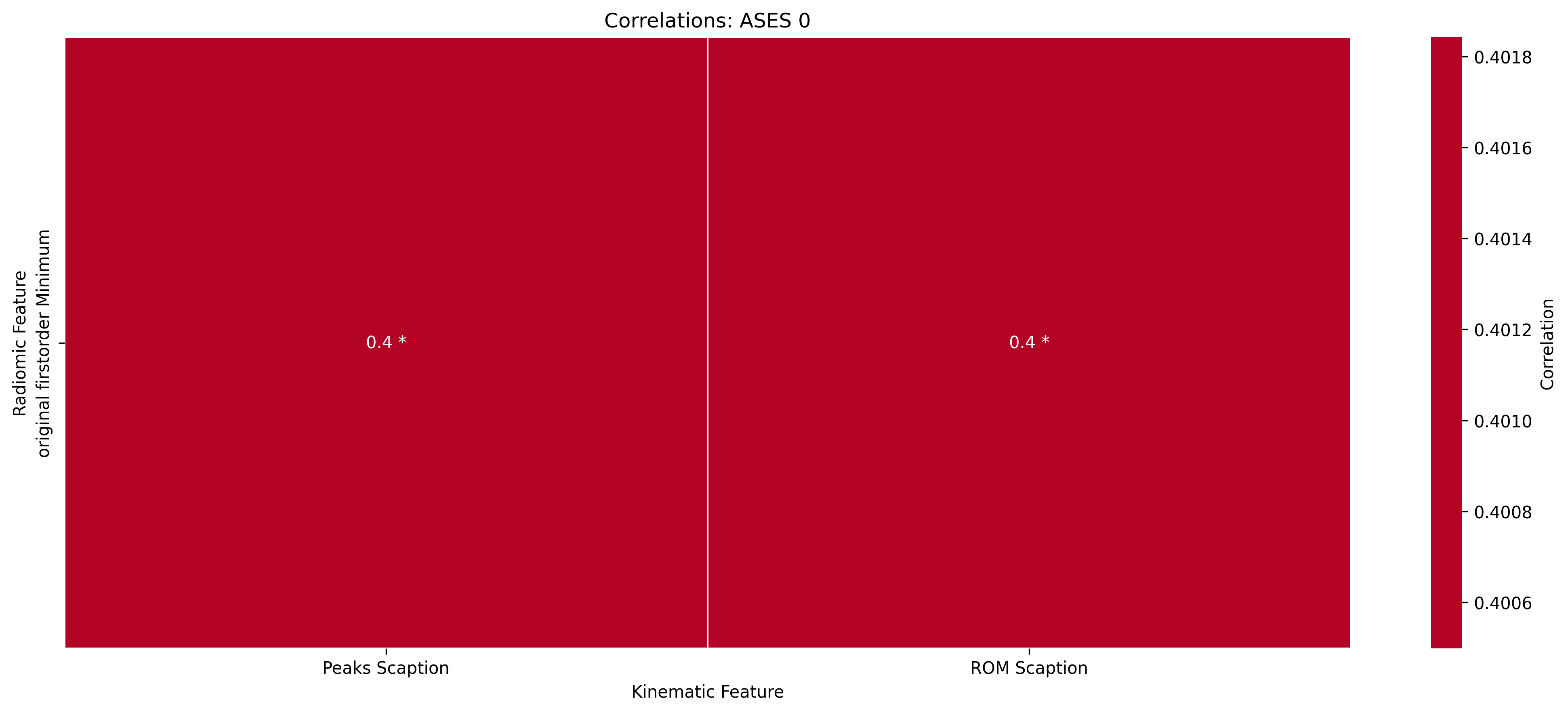

Supplement: Supplementary file 2 [file Image1.TIFF]

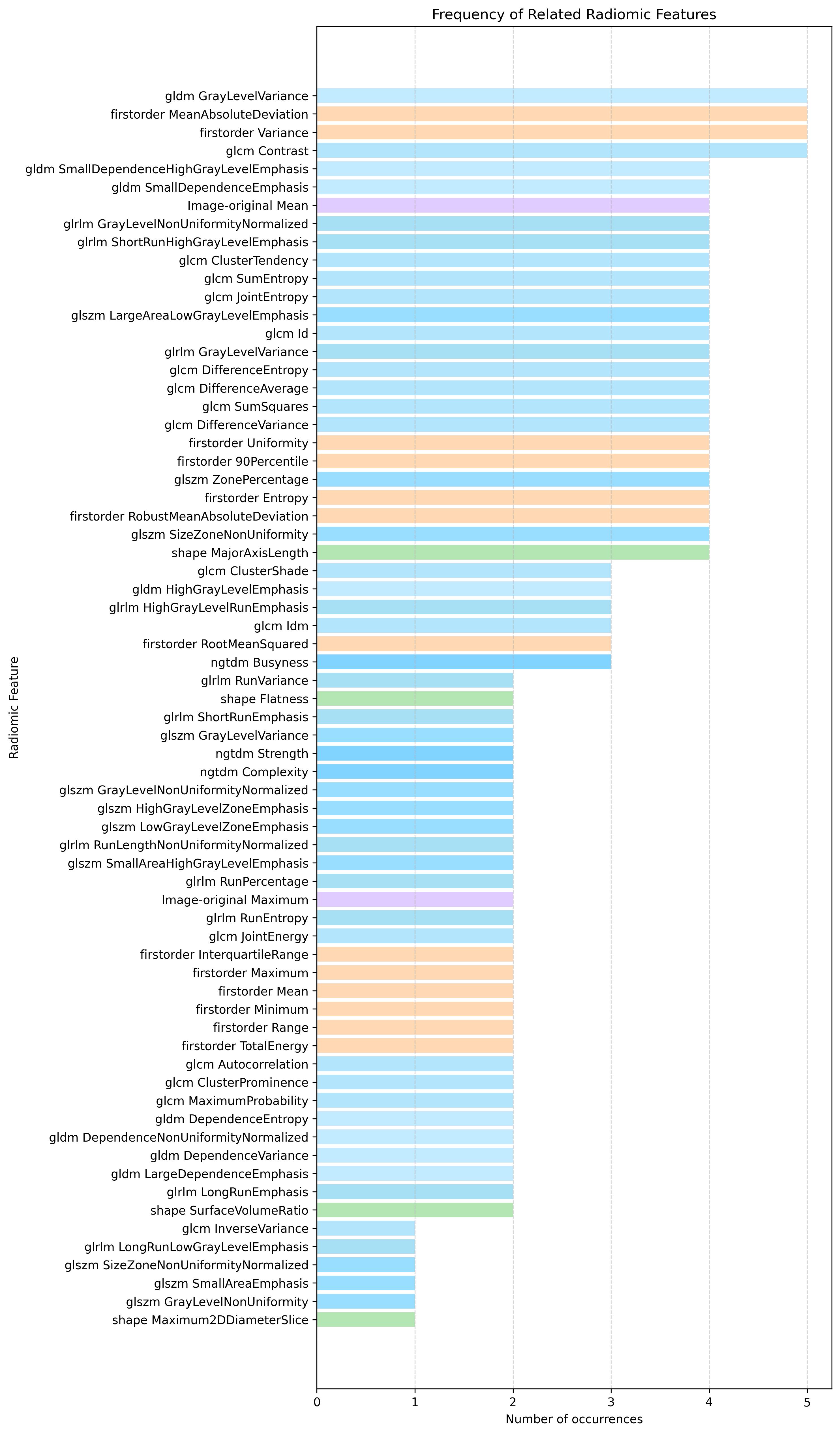

Supplement: Supplementary file 3 [file Image4.JPEG]

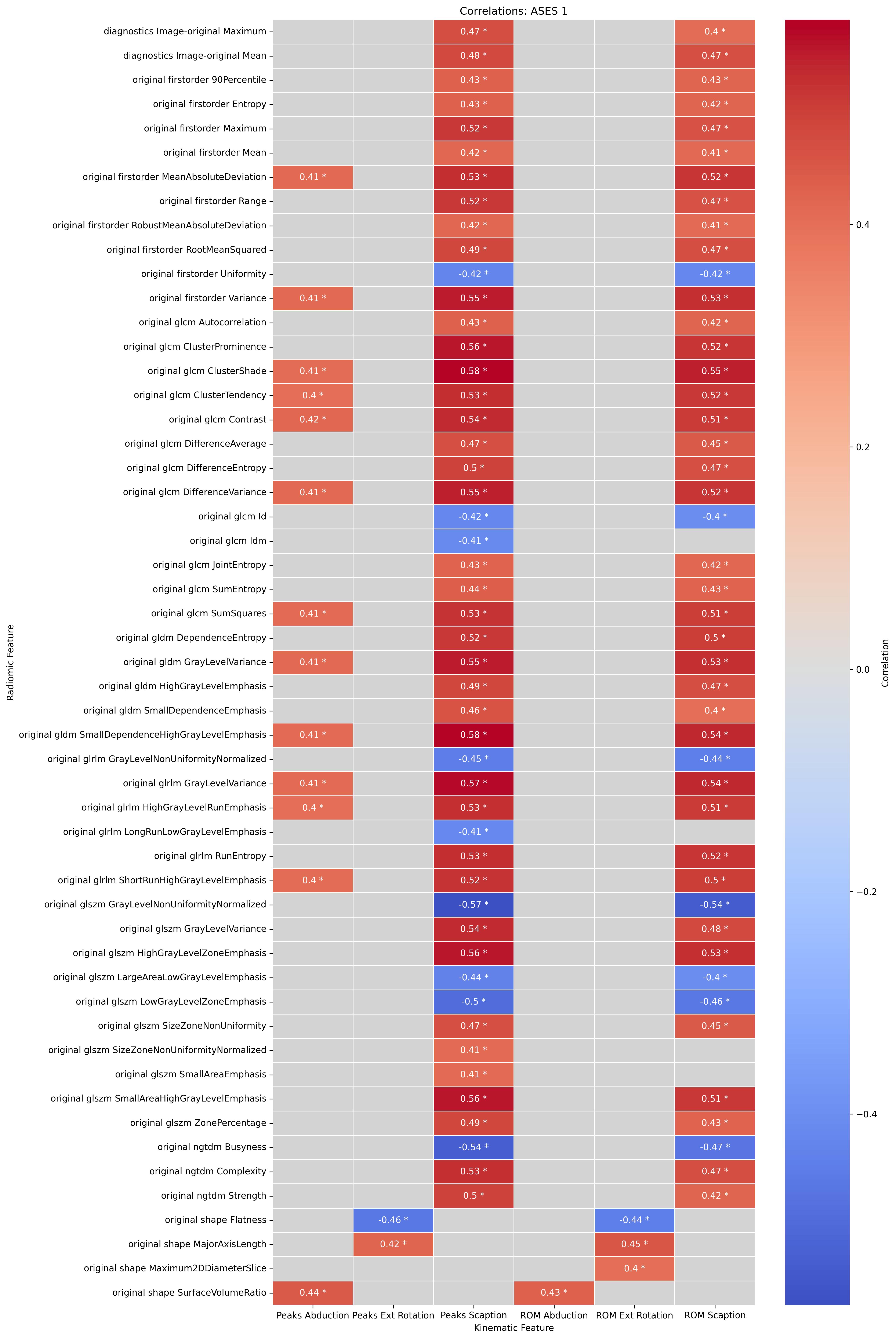

Supplement: Supplementary file 4 [file Image2.JPEG]
