# Supplementary material for: Preoperative radiomic biomarkers reflect functional shoulder impairment in rotator cuff tears: a structure–function analysis
Source: Front Bioeng Biotechnol. 2026 Jul 6;14:1774121. doi: 10.3389/fbioe.2026.1774121 (PMC13381298; doi:10.3389/fbioe.2026.1774121)
Supplement: Supplementary file 5 [file DataSheet1.docx]

Supplementary Material

## Supplementary Figures


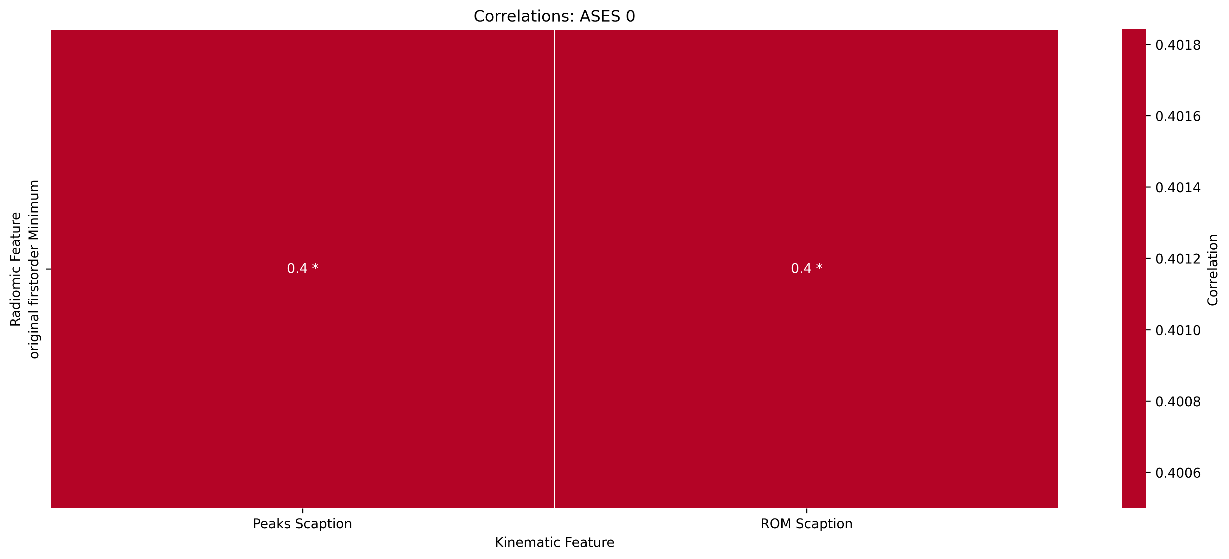


**Supplementary Figure 1.** Heatmap of statistically significant correlations between MRI-derived radiomic features (rows) and kinematic features (columns) for the ASES 0 subgroup. Only statistically significant correlations are shown (*p < 0.05; **p < 0.001).


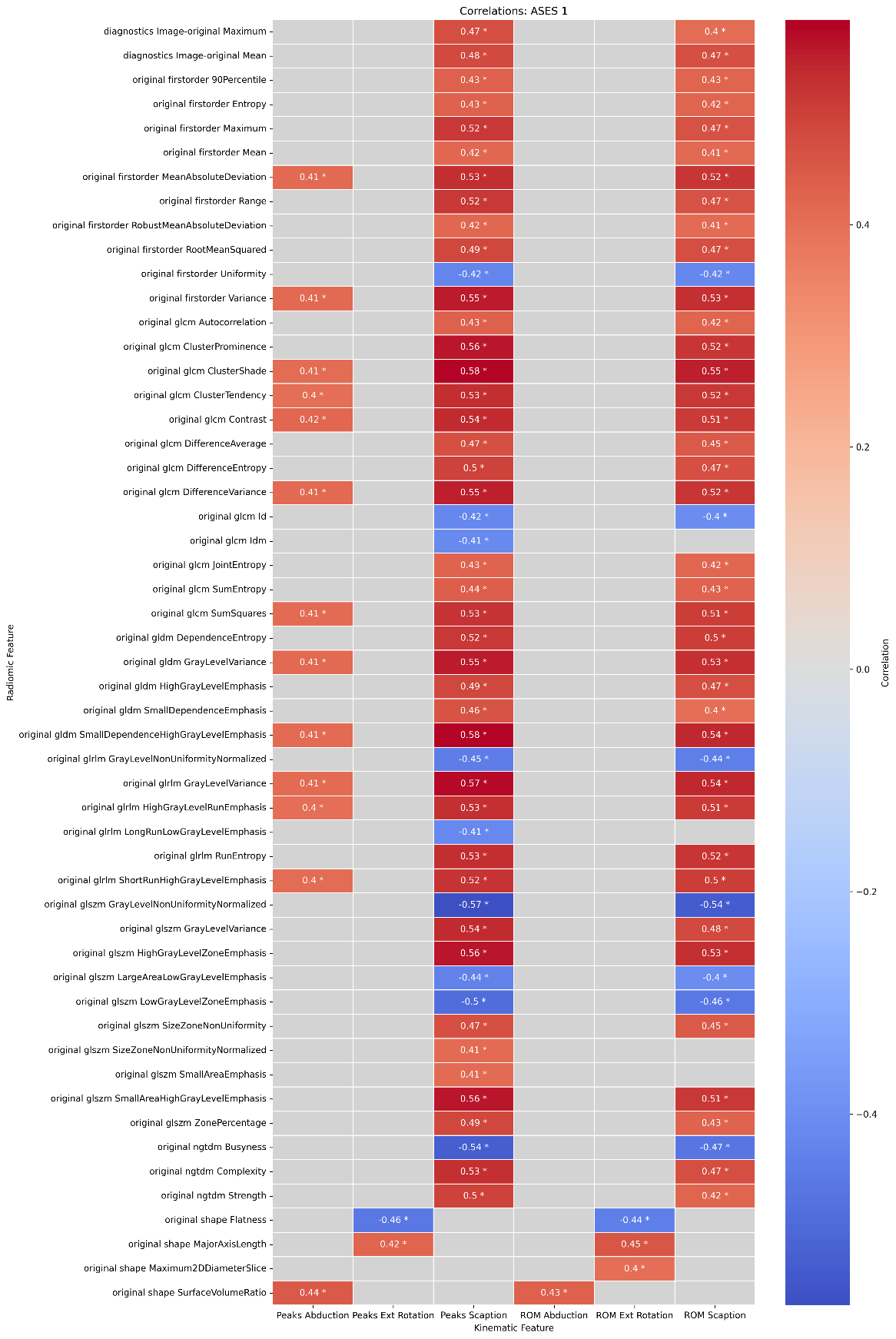


Supplementary Figure 2. Heatmap of statistically significant correlations between MRI-derived radiomic features (rows) and kinematic features (columns) for the ASES 1 subgroup. Color intensity represents the strength and direction of the correlation (red: positive; blue: negative). Only statistically significant correlations are shown (*p < 0.05; **p < 0.001).


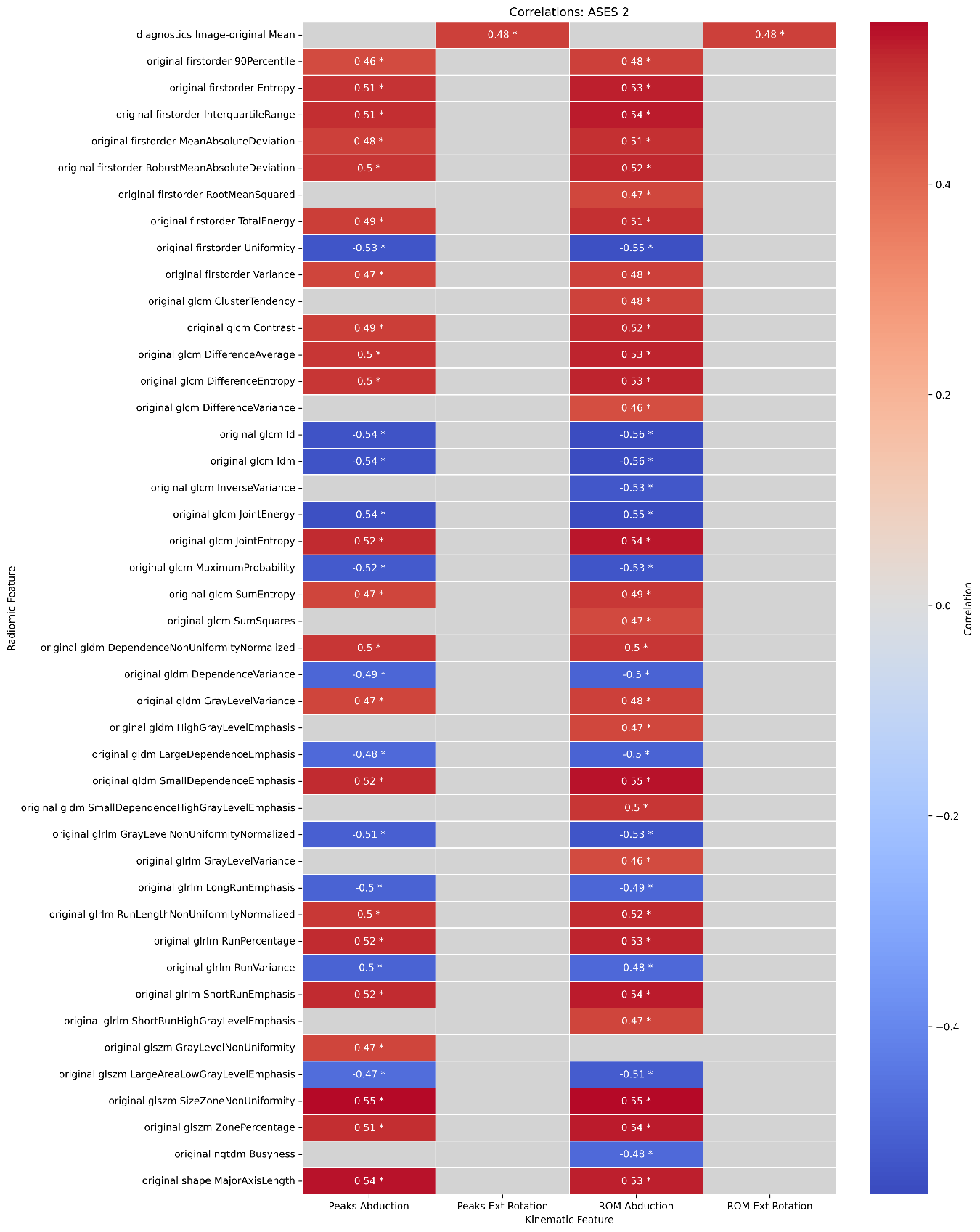


Supplementary Figure 3. Heatmap of statistically significant correlations between MRI-derived radiomic features (rows) and kinematic features (columns) for the ASES 2 subgroup. Color intensity represents the strength and direction of the correlation (red: positive; blue: negative). Only statistically significant correlations are shown (*p < 0.05; **p < 0.001).


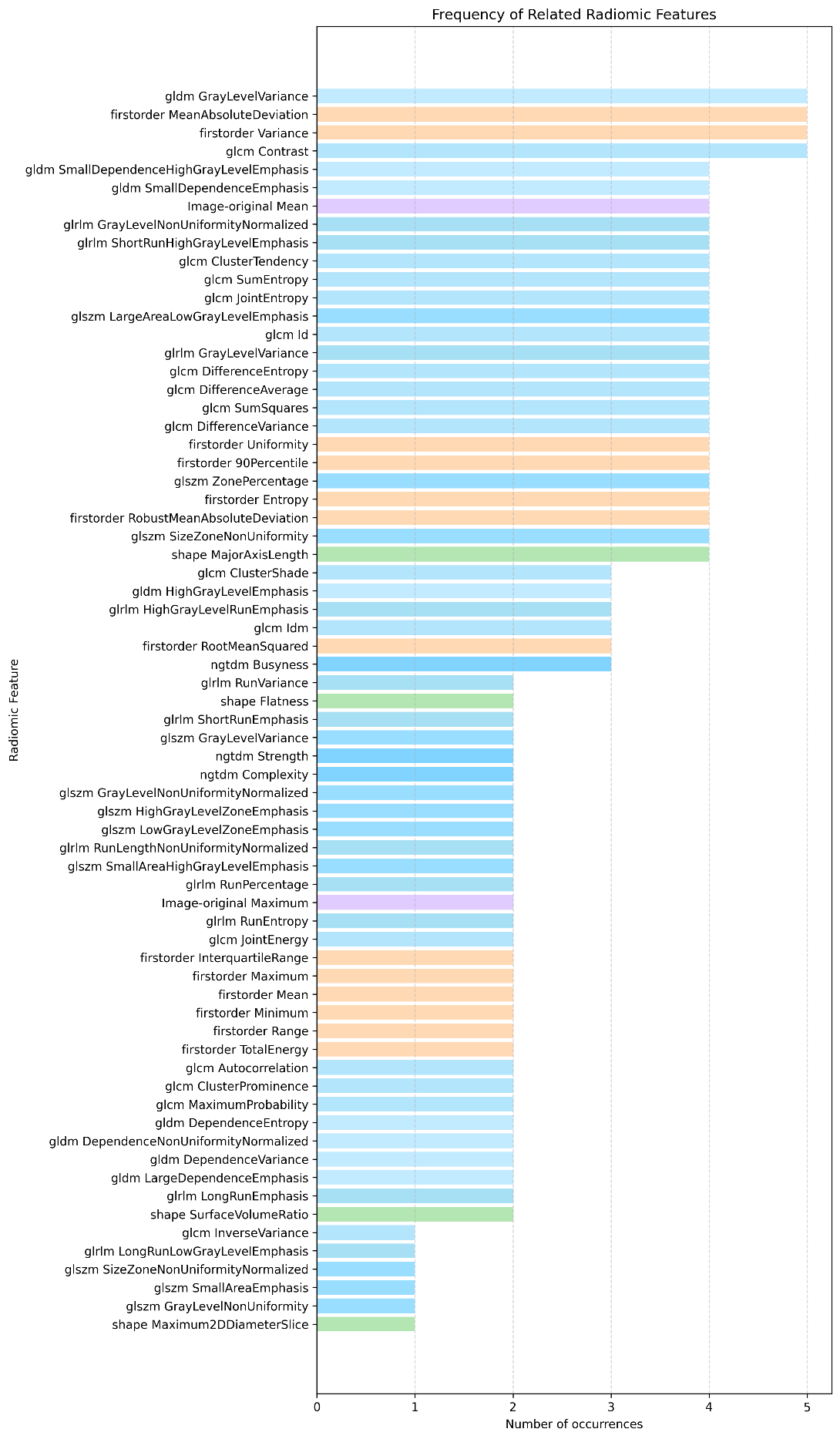


Supplementary Figure 4. Frequency of radiomic features appearing in significant radiomic–kinematic correlations. The bar chart reports the frequency with which each of the 68 radiomic features appeared in statistically significant correlations with kinematic variables. Features are colored according to radiomic category.
